# 6-Bromo-3-{2-[2-(diphenyl­methyl­ene)hydrazin­yl]-1,3-thia­zol-5-yl}-2*H*-chromen-2-one chloro­form monosolvate

**DOI:** 10.1107/S1600536811012335

**Published:** 2011-04-13

**Authors:** Afsheen Arshad, Hasnah Osman, Chan Kit Lam, Madhukar Hemamalini, Hoong-Kun Fun

**Affiliations:** a School of Chemical Sciences, Universiti Sains Malaysia, 11800 USM, Penang, Malaysia; b School of Pharmaceutical Sciences, Universiti Sains Malaysia, 11800 USM, Penang, Malaysia; c X-ray Crystallography Unit, School of Physics, Universiti Sains Malaysia, 11800 USM, Penang, Malaysia

## Abstract

In the title compound, C_25_H_16_BrN_3_O_2_S·CHCl_3_, the thia­zole ring is approximately planar [maximum deviation = 0.002 (3) Å] and makes dihedral angles of 10.75 (14) and 87.75 (15)/2.80 (14)° with the pyran ring system and the two terminal phenyl rings, respectively. The solvent mol­ecule is disordered over two sets of sites, with refined occupancies of 0.639 (7) and 0.361 (7). In the crystal, mol­ecules are connected *via* pairs of weak C—H⋯O inter­actions, forming centrosymmetric dimers. An intra­molecular C—H⋯O hydrogen bond generates an *S*(6) ring motif.

## Related literature

For details and applications of coumarin derivatives, see: Siddiqui *et al.* (2009 ▶); Kamal *et al.* (2009 ▶); Kalkhambkar *et al.* (2007 ▶); Gursoy & Karali (2003 ▶). For the synthesis of benzophenone thio­semicarbazone and 6-bromo-3-(2-bromo­acet­yl)-2*H*-chromen-2-one, see: Yaragatti *et al.* (2010 ▶); Lobana *et al.* (2006 ▶). For graph-set notation, see: Bernstein *et al.* (1995 ▶).
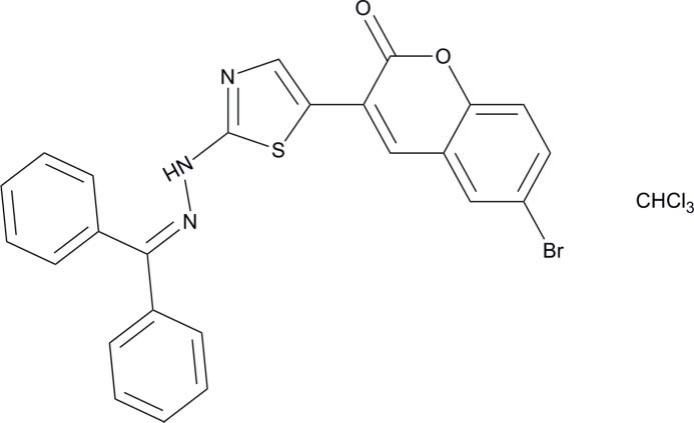


## Experimental

### 

#### Crystal data

C_25_H_16_BrN_3_O_2_S·CHCl_3_
*M* *_r_* = 621.75
Triclinic, 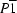

*a* = 8.0774 (3) Å
*b* = 12.6782 (5) Å
*c* = 14.4396 (5) Å
α = 114.157 (2)°
β = 92.879 (2)°
γ = 100.384 (2)°
*V* = 1314.40 (8) Å^3^
*Z* = 2
Mo *K*α radiation
μ = 1.98 mm^−1^
*T* = 296 K
0.56 × 0.14 × 0.06 mm

#### Data collection

Bruker SMART APEXII CCD area-detector diffractometer
Absorption correction: multi-scan (*SADABS*; Bruker, 2009) ▶ *T*_min_ = 0.401, *T*_max_ = 0.891
21464 measured reflections
7663 independent reflections
3262 reflections with *I* > 2σ(*I*)
*R*_int_ = 0.041

#### Refinement

*R*[*F* ^2^ > 2σ(*F* ^2^)] = 0.047
*wR*(*F* ^2^) = 0.125
*S* = 1.00
7663 reflections
357 parameters
H atoms treated by a mixture of independent and constrained refinement
Δρ_max_ = 0.35 e Å^−3^
Δρ_min_ = −0.40 e Å^−3^

### 

Data collection: *APEX2* (Bruker, 2009 ▶); cell refinement: *SAINT* (Bruker, 2009 ▶); data reduction: *SAINT*; program(s) used to solve structure: *SHELXTL* (Sheldrick, 2008 ▶); program(s) used to refine structure: *SHELXTL*; molecular graphics: *SHELXTL*; software used to prepare material for publication: *SHELXTL* and *PLATON* (Spek, 2009 ▶).

## Supplementary Material

Crystal structure: contains datablocks global, I. DOI: 10.1107/S1600536811012335/lh5227sup1.cif

Structure factors: contains datablocks I. DOI: 10.1107/S1600536811012335/lh5227Isup2.hkl

Additional supplementary materials:  crystallographic information; 3D view; checkCIF report

## Figures and Tables

**Table 1:** Hydrogen-bond geometry (Å, °)

*D*—H⋯*A*	*D*—H	H⋯*A*	*D*⋯*A*	*D*—H⋯*A*
C11—H11*A*⋯O2	0.93	2.32	2.868 (4)	117
C19—H19*A*⋯O2^i^	0.93	2.54	3.448 (4)	165
C26—H26*B*⋯O2^ii^	0.96	2.55	3.350 (5)	141

Symmetry codes: (i) 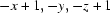; (ii) 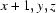.

**Fig. 1.**
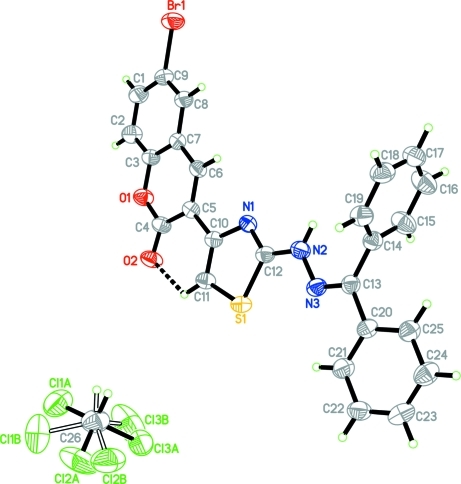
The asymmetric unit of the title compound, showing 30% probability displacement ellipsoids. The intramolecular hydrogen bond is shown as a dashed line. The open bonds represents disordered components.

**Fig. 2.**
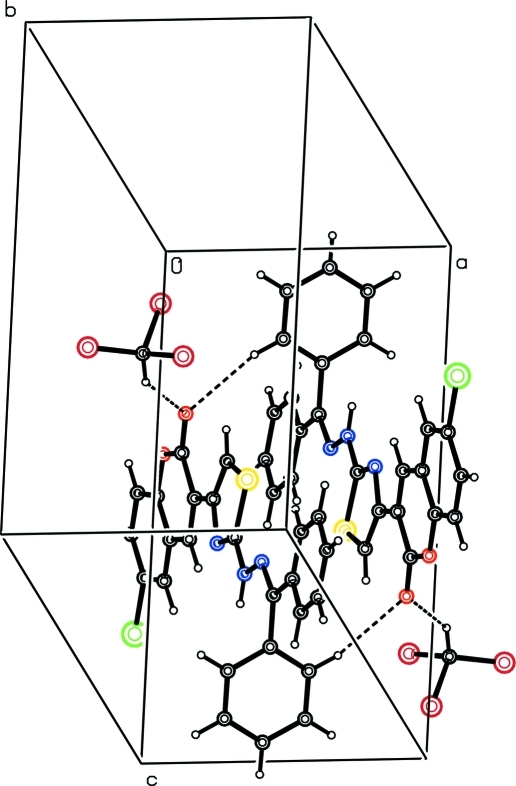
The crystal packing of the title compound with hydrogen bonds shown as dashed lines.

## supplementary crystallographic information

### Comment

The pharmacological properties of coumarin and thiazole rings impart special importance to them in synthetic chemistry. The thiazole incorporated coumarin derivatives exhibit anticonvulsant (Siddiqui *et al.*, 2009), anticancer, antimicrobial (Kamal *et al.*, 2009), analgesic and anti-inflammatory activities (Kalkhambkar *et al.*, 2007). Some of these compounds are also reported to have activity against *Mycobacterium tuberculosis* (Gursoy *et al.*, 2003). The title compound (I) is a new derivative of coumarin having thiazole moiety. We report herein its crystal structure.

The asymmetric unit of the title compound (Fig. 1) consists of one 6-bromo-3-(2-(2-(diphenylmethylene)hydrazinyl)thiazol-5-yl)-2*H*- chromen-2-one molecule and one chloroform solvent molecule.  The thiazole (S1/N1/C10–C12) ring is approximately planar, with maximum deviation of 0.002 (3) Å for atom C10.  The solvent molecule is disordered over two sets of sites, with an occupancy ratio of 0.639 (7): 0.361 (7). The central thiazole (S1/N1/C10–C12) ring makes dihedral angles of  10.75 (12) ° and 87.75 (15) and 2.80 (14)° with the pyran (O1/C3–C7) ring system and the two terminal phenyl (C14–C19/C20–C25) rings, respectively.

In the crystal structure, (Fig. 2), the molecules are connected via weak C19—H19A···O2^i^ and C26—H26B···O2^ii^ (Table 1) interactions. An intramolecular C11—H11A···O2 hydrogen bond generates an *S*(6) ring motif (Bernstein *et al.*, 1995).

### Experimental

Benzophenone thiosemicarbazone (Lobana *et al.*, 2006) and 6-bromo-3-(2-bromoacetyl)-2*H*-chromen-2-one (Yaragatti *et al.*, 2010) were synthesized as reported in the literature. A solution of 6-bromo-3-(2-bromoacetyl)-2*H*-chromen-2-one (2.5 mmol) and benzophenone thiosemicarbazone (2.5 mmol) in chloroform-ethanol (2:1) was refluxed for 1.5 hours. Precipitates formed were filtered and boiled with water containing sodium acetate. The title compound (I) was purified by recrystallization with ethanol-chloroform (1:3) as large brownish, yellow needle-like crystals.

### Refinement

Atom H1N2 was located from a difference Fourier map and refined freely [N–H = 0.87 (3) Å]. The remaining H atoms were positioned geometrically [C–H = 0.93–0.98 Å] and were refined using a riding model, with *U*_iso_(H) = 1.2 *U*_eq_(C).  The solvent molecule is disordered over two sites with a refined occupany ratio of 0.639 (7):0.361 (7).

### Crystal data

**Table:**

C_25_H_16_BrN_3_O_2_S·CHCl_3_	*Z* = 2
*M**_r_* = 621.75	*F*(000) = 624
Triclinic, *P*1	*D*_x_ = 1.571 Mg m^−^^3^
Hall symbol: -P 1	Mo *K*α radiation, λ = 0.71073 Å
*a* = 8.0774 (3) Å	Cell parameters from 4500 reflections
*b* = 12.6782 (5) Å	θ = 2.9–29.6°
*c* = 14.4396 (5) Å	µ = 1.98 mm^−^^1^
α = 114.157 (2)°	*T* = 296 K
β = 92.879 (2)°	Plate, yellow
γ = 100.384 (2)°	0.56 × 0.14 × 0.06 mm
*V* = 1314.40 (8) Å^3^	

### Data collection

**Table:**

Bruker SMART APEXII CCD area-detector diffractometer	7663 independent reflections
Radiation source: fine-focus sealed tube	3262 reflections with *I* > 2σ(*I*)
graphite	*R*_int_ = 0.041
φ and ω scans	θ_max_ = 30.1°, θ_min_ = 1.6°
Absorption correction: multi-scan (*SADABS*; Bruker, 2009)	*h* = −11→11
*T*_min_ = 0.401, *T*_max_ = 0.891	*k* = −17→17
21464 measured reflections	*l* = −20→20

### Refinement

**Table:**

Refinement on *F*^2^	Primary atom site location: structure-invariant direct methods
Least-squares matrix: full	Secondary atom site location: difference Fourier map
*R*[*F*^2^ > 2σ(*F*^2^)] = 0.047	Hydrogen site location: inferred from neighbouring sites
*wR*(*F*^2^) = 0.125	H atoms treated by a mixture of independent and constrained refinement
*S* = 1.00	*w* = 1/[σ^2^(*F*_o_^2^) + (0.0487*P*)^2^ + 0.0043*P*] where *P* = (*F*_o_^2^ + 2*F*_c_^2^)/3
7663 reflections	(Δ/σ)_max_ = 0.001
357 parameters	Δρ_max_ = 0.35 e Å^−^^3^
0 restraints	Δρ_min_ = −0.40 e Å^−^^3^

### Special details

**Table:**

Geometry. All s.u.'s (except the s.u. in the dihedral angle between two l.s. planes) are estimated using the full covariance matrix. The cell s.u.'s are taken into account individually in the estimation of s.u.'s in distances, angles and torsion angles; correlations between s.u.'s in cell parameters are only used when they are defined by crystal symmetry. An approximate (isotropic) treatment of cell s.u.'s is used for estimating s.u.'s involving l.s. planes.
Refinement. Refinement of F^2^ against ALL reflections. The weighted R-factor wR and goodness of fit S are based on F^2^, conventional R-factors R are based on F, with F set to zero for negative F^2^. The threshold expression of F^2^ > 2σ(F^2^) is used only for calculating R-factors(gt) etc. and is not relevant to the choice of reflections for refinement. R-factors based on F^2^ are statistically about twice as large as those based on F, and R- factors based on ALL data will be even larger.

### Fractional atomic coordinates and isotropic or equivalent isotropic displacement parameters (Å^2^)

**Table:**

	*x*	*y*	*z*	*U*_iso_*/*U*_eq_	Occ. (<1)
Br1	−0.38420 (5)	−0.63922 (3)	0.45724 (3)	0.09363 (18)	
S1	0.38790 (9)	0.12055 (6)	0.50298 (6)	0.0610 (2)	
O1	−0.1491 (2)	−0.32668 (16)	0.24872 (13)	0.0643 (5)	
O2	0.0058 (3)	−0.17090 (17)	0.24065 (14)	0.0764 (6)	
N1	0.2137 (3)	−0.03044 (18)	0.55842 (16)	0.0540 (5)	
N2	0.3995 (3)	0.1301 (2)	0.69187 (19)	0.0651 (7)	
N3	0.5130 (3)	0.23407 (18)	0.71595 (17)	0.0591 (6)	
C1	−0.3766 (4)	−0.5695 (3)	0.2940 (2)	0.0740 (8)	
H1A	−0.4596	−0.6386	0.2594	0.089*	
C2	−0.3234 (4)	−0.4974 (2)	0.2462 (2)	0.0726 (8)	
H2A	−0.3701	−0.5172	0.1795	0.087*	
C3	−0.2001 (3)	−0.3959 (2)	0.2988 (2)	0.0568 (7)	
C4	−0.0283 (3)	−0.2219 (2)	0.2938 (2)	0.0582 (7)	
C5	0.0446 (3)	−0.1845 (2)	0.39995 (19)	0.0509 (6)	
C6	−0.0045 (3)	−0.2545 (2)	0.44803 (19)	0.0540 (6)	
H6A	0.0438	−0.2311	0.5152	0.065*	
C7	−0.1285 (3)	−0.3632 (2)	0.39842 (19)	0.0529 (6)	
C8	−0.1827 (4)	−0.4375 (2)	0.4461 (2)	0.0627 (7)	
H8A	−0.1358	−0.4186	0.5126	0.075*	
C9	−0.3070 (4)	−0.5392 (2)	0.3928 (2)	0.0651 (8)	
C10	0.1692 (3)	−0.0710 (2)	0.45345 (19)	0.0505 (6)	
C11	0.2498 (3)	−0.0017 (2)	0.4115 (2)	0.0580 (7)	
H11A	0.2326	−0.0181	0.3423	0.070*	
C12	0.3265 (3)	0.0681 (2)	0.5923 (2)	0.0540 (7)	
C13	0.5912 (3)	0.2922 (2)	0.8078 (2)	0.0549 (6)	
C14	0.5663 (4)	0.2503 (2)	0.8896 (2)	0.0569 (7)	
C15	0.4327 (4)	0.2721 (3)	0.9451 (3)	0.0856 (10)	
H15A	0.3585	0.3140	0.9318	0.103*	
C16	0.4080 (5)	0.2325 (3)	1.0202 (3)	0.0930 (10)	
H16A	0.3189	0.2494	1.0583	0.112*	
C17	0.5125 (5)	0.1691 (3)	1.0390 (2)	0.0788 (9)	
H17A	0.4941	0.1414	1.0889	0.095*	
C18	0.6456 (4)	0.1458 (3)	0.9841 (2)	0.0775 (9)	
H18A	0.7180	0.1026	0.9970	0.093*	
C19	0.6724 (4)	0.1868 (2)	0.9093 (2)	0.0690 (8)	
H19A	0.7630	0.1709	0.8723	0.083*	
C20	0.7132 (3)	0.4042 (2)	0.8283 (2)	0.0567 (7)	
C21	0.7536 (4)	0.4354 (2)	0.7496 (2)	0.0673 (8)	
H21A	0.7037	0.3853	0.6828	0.081*	
C22	0.8674 (4)	0.5400 (3)	0.7692 (3)	0.0784 (9)	
H22A	0.8936	0.5599	0.7155	0.094*	
C23	0.9421 (4)	0.6150 (3)	0.8679 (3)	0.0815 (9)	
H23A	1.0189	0.6853	0.8811	0.098*	
C24	0.9026 (4)	0.5853 (3)	0.9461 (3)	0.0789 (9)	
H24A	0.9521	0.6361	1.0130	0.095*	
C25	0.7901 (4)	0.4809 (2)	0.9271 (2)	0.0683 (8)	
H25A	0.7654	0.4615	0.9812	0.082*	
C26	0.9833 (5)	0.0897 (3)	0.2392 (3)	0.0975 (11)	
H26A	0.9796	0.0622	0.2935	0.117*	0.372 (7)
H26B	0.9696	0.0357	0.2707	0.117*	0.628 (7)
Cl1A	0.8630 (12)	−0.0189 (3)	0.1341 (3)	0.136 (3)	0.372 (7)
Cl2A	1.1856 (9)	0.1392 (9)	0.2352 (8)	0.157 (3)	0.372 (7)
Cl3A	0.8588 (9)	0.2080 (4)	0.2724 (5)	0.0986 (14)	0.372 (7)
Cl1B	0.9957 (6)	0.0084 (4)	0.1053 (2)	0.1538 (16)	0.628 (7)
Cl2B	1.1788 (7)	0.1888 (3)	0.2910 (3)	0.1360 (14)	0.628 (7)
Cl3B	0.8225 (10)	0.1494 (8)	0.2524 (5)	0.213 (3)	0.628 (7)
H1N2	0.384 (4)	0.102 (3)	0.737 (2)	0.091 (11)*	

### Atomic displacement parameters (Å^2^)

**Table:**

	*U*^11^	*U*^22^	*U*^33^	*U*^12^	*U*^13^	*U*^23^
Br1	0.1128 (3)	0.0744 (2)	0.0971 (3)	−0.0036 (2)	0.0211 (2)	0.0490 (2)
S1	0.0596 (5)	0.0589 (4)	0.0710 (5)	0.0057 (3)	0.0107 (4)	0.0367 (4)
O1	0.0655 (12)	0.0705 (12)	0.0572 (11)	0.0013 (10)	−0.0032 (9)	0.0344 (9)
O2	0.0841 (15)	0.0871 (13)	0.0646 (12)	−0.0019 (11)	0.0008 (10)	0.0483 (11)
N1	0.0526 (13)	0.0562 (12)	0.0549 (14)	0.0093 (11)	0.0099 (11)	0.0263 (11)
N2	0.0742 (17)	0.0621 (14)	0.0549 (16)	0.0001 (13)	0.0053 (13)	0.0272 (13)
N3	0.0565 (14)	0.0509 (12)	0.0676 (16)	0.0045 (11)	0.0083 (12)	0.0261 (11)
C1	0.073 (2)	0.0605 (17)	0.079 (2)	−0.0024 (15)	0.0021 (17)	0.0286 (16)
C2	0.071 (2)	0.0718 (18)	0.0674 (19)	0.0002 (17)	−0.0081 (16)	0.0311 (16)
C3	0.0519 (16)	0.0585 (15)	0.0644 (18)	0.0090 (14)	0.0076 (14)	0.0320 (14)
C4	0.0489 (17)	0.0678 (17)	0.0636 (18)	0.0086 (14)	0.0065 (14)	0.0356 (15)
C5	0.0444 (15)	0.0606 (15)	0.0518 (15)	0.0123 (13)	0.0079 (12)	0.0275 (13)
C6	0.0555 (16)	0.0586 (15)	0.0479 (15)	0.0086 (13)	0.0062 (12)	0.0244 (13)
C7	0.0537 (16)	0.0541 (14)	0.0518 (16)	0.0113 (13)	0.0104 (13)	0.0235 (12)
C8	0.0709 (19)	0.0566 (16)	0.0599 (17)	0.0080 (15)	0.0134 (15)	0.0262 (14)
C9	0.071 (2)	0.0552 (16)	0.072 (2)	0.0083 (15)	0.0191 (16)	0.0304 (15)
C10	0.0460 (15)	0.0539 (14)	0.0576 (17)	0.0125 (12)	0.0084 (12)	0.0289 (13)
C11	0.0575 (17)	0.0646 (16)	0.0598 (16)	0.0117 (13)	0.0091 (13)	0.0348 (14)
C12	0.0498 (16)	0.0543 (15)	0.0647 (18)	0.0134 (13)	0.0140 (14)	0.0304 (14)
C13	0.0546 (16)	0.0530 (14)	0.0573 (17)	0.0130 (13)	0.0086 (14)	0.0230 (13)
C14	0.0532 (17)	0.0545 (15)	0.0578 (17)	0.0042 (14)	0.0013 (14)	0.0225 (13)
C15	0.083 (2)	0.113 (3)	0.090 (2)	0.043 (2)	0.030 (2)	0.062 (2)
C16	0.090 (3)	0.124 (3)	0.089 (2)	0.040 (2)	0.037 (2)	0.060 (2)
C17	0.092 (3)	0.078 (2)	0.068 (2)	0.0045 (19)	0.0092 (19)	0.0389 (17)
C18	0.088 (3)	0.0714 (19)	0.083 (2)	0.0185 (18)	0.005 (2)	0.0433 (17)
C19	0.0654 (19)	0.0707 (18)	0.080 (2)	0.0171 (16)	0.0148 (16)	0.0398 (17)
C20	0.0539 (17)	0.0526 (15)	0.0653 (18)	0.0127 (13)	0.0068 (14)	0.0266 (14)
C21	0.069 (2)	0.0631 (17)	0.0687 (19)	0.0103 (15)	0.0092 (15)	0.0289 (15)
C22	0.079 (2)	0.0716 (19)	0.093 (2)	0.0081 (17)	0.0210 (19)	0.0460 (19)
C23	0.074 (2)	0.0603 (18)	0.107 (3)	0.0039 (16)	0.006 (2)	0.038 (2)
C24	0.071 (2)	0.0663 (19)	0.086 (2)	0.0033 (17)	−0.0089 (17)	0.0267 (18)
C25	0.073 (2)	0.0603 (17)	0.071 (2)	0.0127 (16)	0.0038 (16)	0.0290 (16)
C26	0.118 (3)	0.095 (2)	0.098 (3)	0.022 (2)	−0.002 (2)	0.063 (2)
Cl1A	0.211 (7)	0.0912 (19)	0.097 (3)	0.014 (3)	−0.030 (3)	0.0435 (17)
Cl2A	0.108 (4)	0.223 (8)	0.229 (8)	0.052 (5)	0.074 (5)	0.171 (7)
Cl3A	0.117 (3)	0.107 (2)	0.098 (2)	0.055 (2)	0.0259 (19)	0.055 (2)
Cl1B	0.160 (3)	0.148 (2)	0.1210 (18)	0.065 (2)	0.0162 (18)	0.0132 (15)
Cl2B	0.174 (3)	0.1110 (18)	0.128 (2)	−0.0059 (17)	−0.007 (2)	0.0741 (17)
Cl3B	0.280 (6)	0.332 (7)	0.185 (4)	0.231 (6)	0.132 (4)	0.190 (5)

### Geometric parameters (Å, °)

**Table:**

Br1—C9	1.898 (3)	C14—C19	1.370 (4)
S1—C11	1.716 (3)	C14—C15	1.377 (4)
S1—C12	1.732 (2)	C15—C16	1.379 (4)
O1—C3	1.372 (3)	C15—H15A	0.9300
O1—C4	1.379 (3)	C16—C17	1.353 (4)
O2—C4	1.202 (3)	C16—H16A	0.9300
N1—C12	1.295 (3)	C17—C18	1.371 (4)
N1—C10	1.390 (3)	C17—H17A	0.9300
N2—C12	1.362 (3)	C18—C19	1.387 (4)
N2—N3	1.363 (3)	C18—H18A	0.9300
N2—H1N2	0.87 (3)	C19—H19A	0.9300
N3—C13	1.284 (3)	C20—C21	1.382 (4)
C1—C9	1.375 (4)	C20—C25	1.387 (4)
C1—C2	1.378 (4)	C21—C22	1.383 (4)
C1—H1A	0.9300	C21—H21A	0.9300
C2—C3	1.373 (3)	C22—C23	1.378 (4)
C2—H2A	0.9300	C22—H22A	0.9300
C3—C7	1.384 (4)	C23—C24	1.364 (4)
C4—C5	1.461 (3)	C23—H23A	0.9300
C5—C6	1.353 (3)	C24—C25	1.378 (4)
C5—C10	1.473 (3)	C24—H24A	0.9300
C6—C7	1.428 (3)	C25—H25A	0.9300
C6—H6A	0.9300	C26—Cl3B	1.599 (7)
C7—C8	1.402 (3)	C26—Cl2A	1.655 (8)
C8—C9	1.381 (4)	C26—Cl1A	1.674 (5)
C8—H8A	0.9300	C26—Cl2B	1.740 (6)
C10—C11	1.354 (3)	C26—Cl1B	1.798 (5)
C11—H11A	0.9300	C26—Cl3A	1.869 (7)
C13—C20	1.483 (3)	C26—H26A	0.9800
C13—C14	1.488 (3)	C26—H26B	0.9601
			
C11—S1—C12	87.98 (13)	C16—C17—C18	119.8 (3)
C3—O1—C4	122.8 (2)	C16—C17—H17A	120.1
C12—N1—C10	109.2 (2)	C18—C17—H17A	120.1
C12—N2—N3	117.0 (2)	C17—C18—C19	120.0 (3)
C12—N2—H1N2	122 (2)	C17—C18—H18A	120.0
N3—N2—H1N2	120 (2)	C19—C18—H18A	120.0
C13—N3—N2	118.9 (2)	C14—C19—C18	120.4 (3)
C9—C1—C2	120.1 (3)	C14—C19—H19A	119.8
C9—C1—H1A	119.9	C18—C19—H19A	119.8
C2—C1—H1A	119.9	C21—C20—C25	118.1 (2)
C3—C2—C1	118.8 (3)	C21—C20—C13	121.0 (2)
C3—C2—H2A	120.6	C25—C20—C13	120.9 (2)
C1—C2—H2A	120.6	C20—C21—C22	120.8 (3)
O1—C3—C2	117.3 (2)	C20—C21—H21A	119.6
O1—C3—C7	120.5 (2)	C22—C21—H21A	119.6
C2—C3—C7	122.2 (2)	C23—C22—C21	120.2 (3)
O2—C4—O1	115.8 (2)	C23—C22—H22A	119.9
O2—C4—C5	126.9 (2)	C21—C22—H22A	119.9
O1—C4—C5	117.3 (2)	C24—C23—C22	119.5 (3)
C6—C5—C4	119.4 (2)	C24—C23—H23A	120.3
C6—C5—C10	120.9 (2)	C22—C23—H23A	120.3
C4—C5—C10	119.7 (2)	C23—C24—C25	120.6 (3)
C5—C6—C7	121.7 (2)	C23—C24—H24A	119.7
C5—C6—H6A	119.1	C25—C24—H24A	119.7
C7—C6—H6A	119.1	C24—C25—C20	120.8 (3)
C3—C7—C8	118.5 (2)	C24—C25—H25A	119.6
C3—C7—C6	118.3 (2)	C20—C25—H25A	119.6
C8—C7—C6	123.2 (2)	Cl3B—C26—Cl2A	132.4 (4)
C9—C8—C7	118.9 (3)	Cl3B—C26—Cl1A	85.1 (3)
C9—C8—H8A	120.5	Cl2A—C26—Cl1A	119.6 (4)
C7—C8—H8A	120.5	Cl3B—C26—Cl2B	115.0 (4)
C1—C9—C8	121.4 (2)	Cl2A—C26—Cl2B	27.3 (3)
C1—C9—Br1	119.3 (2)	Cl1A—C26—Cl2B	146.5 (4)
C8—C9—Br1	119.3 (2)	Cl3B—C26—Cl1B	110.0 (3)
C11—C10—N1	115.4 (2)	Cl2A—C26—Cl1B	79.3 (4)
C11—C10—C5	127.3 (2)	Cl1A—C26—Cl1B	40.7 (2)
N1—C10—C5	117.4 (2)	Cl2B—C26—Cl1B	105.8 (3)
C10—C11—S1	110.9 (2)	Cl3B—C26—Cl3A	20.7 (4)
C10—C11—H11A	124.6	Cl2A—C26—Cl3A	112.6 (4)
S1—C11—H11A	124.6	Cl1A—C26—Cl3A	102.1 (4)
N1—C12—N2	124.1 (2)	Cl2B—C26—Cl3A	94.4 (3)
N1—C12—S1	116.6 (2)	Cl1B—C26—Cl3A	116.5 (3)
N2—C12—S1	119.33 (19)	Cl3B—C26—H26A	101.7
N3—C13—C20	116.0 (2)	Cl2A—C26—H26A	107.3
N3—C13—C14	123.1 (2)	Cl1A—C26—H26A	107.3
C20—C13—C14	120.9 (2)	Cl2B—C26—H26A	95.1
C19—C14—C15	118.7 (3)	Cl1B—C26—H26A	129.1
C19—C14—C13	120.9 (3)	Cl3A—C26—H26A	107.3
C15—C14—C13	120.4 (3)	Cl3B—C26—H26B	108.6
C14—C15—C16	120.6 (3)	Cl2A—C26—H26B	112.0
C14—C15—H15A	119.7	Cl1A—C26—H26B	88.0
C16—C15—H15A	119.7	Cl2B—C26—H26B	108.5
C17—C16—C15	120.5 (3)	Cl1B—C26—H26B	108.8
C17—C16—H16A	119.8	Cl3A—C26—H26B	120.5
C15—C16—H16A	119.8	H26A—C26—H26B	20.9
			
C12—N2—N3—C13	−176.4 (2)	C12—S1—C11—C10	0.2 (2)
C9—C1—C2—C3	0.2 (5)	C10—N1—C12—N2	−178.7 (2)
C4—O1—C3—C2	179.0 (3)	C10—N1—C12—S1	0.3 (3)
C4—O1—C3—C7	−1.1 (4)	N3—N2—C12—N1	−178.3 (2)
C1—C2—C3—O1	179.5 (3)	N3—N2—C12—S1	2.8 (3)
C1—C2—C3—C7	−0.4 (5)	C11—S1—C12—N1	−0.3 (2)
C3—O1—C4—O2	179.1 (2)	C11—S1—C12—N2	178.7 (2)
C3—O1—C4—C5	−0.8 (4)	N2—N3—C13—C20	179.8 (2)
O2—C4—C5—C6	−178.0 (3)	N2—N3—C13—C14	1.1 (4)
O1—C4—C5—C6	1.9 (4)	N3—C13—C14—C19	94.4 (3)
O2—C4—C5—C10	2.5 (4)	C20—C13—C14—C19	−84.2 (3)
O1—C4—C5—C10	−177.6 (2)	N3—C13—C14—C15	−83.9 (4)
C4—C5—C6—C7	−1.2 (4)	C20—C13—C14—C15	97.5 (3)
C10—C5—C6—C7	178.3 (2)	C19—C14—C15—C16	1.1 (5)
O1—C3—C7—C8	−179.1 (2)	C13—C14—C15—C16	179.3 (3)
C2—C3—C7—C8	0.8 (4)	C14—C15—C16—C17	−1.5 (5)
O1—C3—C7—C6	1.8 (4)	C15—C16—C17—C18	1.1 (5)
C2—C3—C7—C6	−178.3 (3)	C16—C17—C18—C19	−0.3 (5)
C5—C6—C7—C3	−0.6 (4)	C15—C14—C19—C18	−0.3 (4)
C5—C6—C7—C8	−179.7 (3)	C13—C14—C19—C18	−178.5 (3)
C3—C7—C8—C9	−1.0 (4)	C17—C18—C19—C14	−0.1 (4)
C6—C7—C8—C9	178.1 (3)	N3—C13—C20—C21	−7.5 (4)
C2—C1—C9—C8	−0.4 (5)	C14—C13—C20—C21	171.2 (3)
C2—C1—C9—Br1	179.4 (2)	N3—C13—C20—C25	172.5 (3)
C7—C8—C9—C1	0.8 (4)	C14—C13—C20—C25	−8.8 (4)
C7—C8—C9—Br1	−179.0 (2)	C25—C20—C21—C22	0.0 (4)
C12—N1—C10—C11	−0.1 (3)	C13—C20—C21—C22	179.9 (3)
C12—N1—C10—C5	178.5 (2)	C20—C21—C22—C23	−0.1 (5)
C6—C5—C10—C11	168.7 (3)	C21—C22—C23—C24	−0.2 (5)
C4—C5—C10—C11	−11.8 (4)	C22—C23—C24—C25	0.5 (5)
C6—C5—C10—N1	−9.7 (4)	C23—C24—C25—C20	−0.7 (5)
C4—C5—C10—N1	169.8 (2)	C21—C20—C25—C24	0.4 (4)
N1—C10—C11—S1	−0.1 (3)	C13—C20—C25—C24	−179.6 (3)
C5—C10—C11—S1	−178.5 (2)		

### Hydrogen-bond geometry (Å, °)

**Table:**

*D*—H···*A*	*D*—H	H···*A*	*D*···*A*	*D*—H···*A*
C11—H11A···O2	0.93	2.32	2.868 (4)	117
C19—H19A···O2^i^	0.93	2.54	3.448 (4)	165
C26—H26B···O2^ii^	0.96	2.55	3.350 (5)	141

Symmetry codes: (i) −*x*+1, −*y*, −*z*+1; (ii) *x*+1, *y*, *z*.
